# Musculoskeletal pain among medical residents: role of workplace safety climate and sexual harassment

**DOI:** 10.1186/s12891-024-07272-w

**Published:** 2024-02-22

**Authors:** Maha E. Ibrahim, Safaa M. El-Zoghby, Nancy M. Zaghloul, Shaimaa A. Shehata, Rasha M. Farghaly

**Affiliations:** 1 Department of Physical Medicine, Rheumatology and Rehabilitation, Faculty of Medicine, Suez Canal University, Ismailia, 41522 Egypt; 2 Department of Family Medicine, Faculty of Medicine, Suez Canal University, Ismailia, 41522 Egypt; 3 Department of Forensic Medicine and Clinical Toxicology, Misr University for Science and Technology, Cairo, Egypt; 4 Department of Forensic Medicine and Clinical Toxicology, Faculty of Medicine, Suez Canal University, Ismailia, 41522 Egypt; 5 Department of Community, Occupational and Environmental Medicine, Faculty of Medicine, Suez Canal University, Ismailia, 41522 Egypt

**Keywords:** Medical residents, Musculoskeletal pain, Sexual harassment, Workplace violence

## Abstract

**Background:**

Workplace factors are important predictors of occurrence of musculoskeletal pain among different occupational populations. In healthcare, a psychologically unsafe work environment can negatively affect the emotional, physical and psychological well-being of physicians. This study aimed to examine the relationship between workplace violence, sexual harassment and musculoskeletal pain among Egyptian physicians in their years of residency.

**Methods:**

We distributed an online self-administered questionnaire to 101 residents working in various healthcare sectors in Egypt. It included sections on demographic data, working conditions, widespread pain index (WPI), pain interference short-form, workplace violence and harassment questionnaire, psychosocial safety climate questionnaire (PSC) and sexual harassment climate questionnaire.

**Results:**

All residents had at least one painful site on the WPI (range 1–11). The mean WPI was 3.5 ± 2.4, and 39.6% satisfied the criteria of having widespread pain by having at least 4 pain sites. Widespread pain index showed a weak statistically significant negative correlation with workplace PSC score (rho = − 0.272, *p* = 0.006), and a statistically significant weak positive correlation with the calculated total abuse index (rho = 0.305, *p* = 0.002). Workplace violence and abuse, as measured by a calculated abuse index was the only significant predictors of widespread pain among residents.

**Conclusion:**

WPV was found to be a predictor of musculoskeletal pain among medical residents. Healthcare organizations need to address WPV by employing preventive strategies to minimize its hazardous effects and ensure a safe working environment for physicians.

## Background

Musculoskeletal pain is a common symptom among healthcare workers, including physicians. Several studies have reported a high prevalence of musculoskeletal pain among physicians across different specialties [1–5]. Various workplace risk factors have been implicated in the occurrence of musculoskeletal pain among healthcare workers. These include ergonomic factors and physical job demands [6, 7], long working hours and night shifts [8], psychosocial and emotional burdens of the profession [3] and perceptions about workplace support [7]. A recent study conducted in Egypt found the prevalence of musculoskeletal disorders among physicians to be as high as 74% [9]. In addition to the previously mentioned factors, workplace violence (WPV) seems to play a role in the occurrence of musculoskeletal pain among healthcare workers [10]. A large survey in United States among nurses revealed that the prevalence of low back pain was as high as 70% among those who experienced WPV, compared to 40% among non-victimized workers [11].

Workplace violence (WPV) is a distinguished global occupational hazard that faces workers in the health care system, whether from the patients or their relatives, or even from other physicians and co-worker [12, 13]. The International Labour Organization defines WPV as “a range of unacceptable behaviours and practices, or threats thereof, that aim at, result in, or are likely to result in physical, psychological, sexual or economic harm…”. WPV also encompasses gender-based violence and harassment, which means violence and harassment directed at persons because of their sex or gender, or affecting persons of a particular sex or gender disproportionately [14].

Nearly all forms of WPV are prevalent among physicians. For example, a systematic review in 2019 reported that the overall prevalence of various types of WPV (physical, verbal and sexual) among physicians was 69%; 38% of them were classified as severe forms of violence [13]. In the same line, a study conducted in the United States found that 20 to 30% of the physicians endured racially and sexist offensive remarks, or unwanted sexual intimidations by patients, their relatives and other visitors [15]. It was noted that violence against healthcare workers has increased during the COVID-19 pandemic and has been increasing still since then [16, 17]. A recent study conducted in Egypt reported that approximately 43% of physicians were victims of psychological violence and 10% were victims of physical violence during the COVID-19 pandemic. The study also reported that the majority of physicians did not report the violent incidents [18].

Workplace violence is a significant problem worldwide, and more so in developing countries. A systematic review on WPV in African countries found the prevalence of WPV against physicians to range from 9 to 100%, where the highest reports were from South Africa (54–100%) and Egypt (60–86%) [19]. The study also reported that most African countries lacked policies addressing management strategies. Another Egyptian study indicated that about 80% of physicians who reported incidents of WPV were not satisfied with the actions taken by the authorities [20]. Despite the fact that the Egyptian law penalizes WPV against government officials, including healthcare workers, lack of polices at the institutional level, fear of punishment, and long legal procedures maintain low reporting rates [21, 22].

WPV can lead to profound negative consequences for physicians including burnout, depression, increasing anxiety, and emotional distress [23, 24]. In addition, it affects the quality of service and was found to motivate early retiring from work [25, 26]. Surprisingly, this could happen not just to the victimized physicians, but also to their co-workers who are in-directly assaulted [27]. In addition to mental health issues, previous studies have mentioned the occurrence of physical symptoms as a response to WPV [22, 28]. One of the reported physical symptoms by victims of violence is musculoskeletal pain. Studies have shown that most of those who are being repeatedly violated suffered moderate to severe pain, where the pain in more than half of them affected their work and/or sleep. Moreover, the musculoskeletal pain outcomes were 1.5 to 2.5 times more frequent in those subjected frequently to physical violence than their colleagues who were not [10].

Despite the high prevalence of musculoskeletal pain and WPV among physicians, few studies have examined the relation between the two phenomena. Additionally, the local context in Egypt represents a unique context, and thus warrants further investigation. For example, in the last few years, an alarming number of Egyptian residents have emigrated to other countries. The Egyptian Medical Syndicate reports that Egypt has lost more than 5% of its working physicians in a period of 3 years (2015–2018) [29]. Among the factors that were listed as negative motivators, were verbal and physical assault of physicians [30]. As mentioned previously, estimates of WPV among healthcare workers in Egypt range from 27 to 86% [18, 19, 31, 32]. Therefore, we conducted this study to investigate the relationship between workplace violence, including sexual harassment, and musculoskeletal pain among Egyptian medical residents. We also aimed to explore the association between musculoskeletal pain and different demographic and work-related determinants.

## Methods

This was a cross-sectional survey study on medical residents working in the private and public sector hospitals in Egypt. We used an online self-administered structured questionnaire. We shared the questionnaire on social media platforms that are dedicated to residents all over Egypt. An informed consent form was included on the first page of the online survey for all participants. Participants were directed to the questionnaire after agreeing to participate. Proceeding beyond the first page implied consent.

### Participants and procedure

We recruited physicians in their residency training years who work in public and private Egyptian Hospitals. Public hospitals included university hospitals as well as hospitals affiliated to the Egyptian ministry of health and population. We used convenience sampling technique, since it was not possible to obtain a sampling frame. An online survey was disseminated through a link shared on different social media platforms from March 2021 to August 2021. The platforms included Facebook and Whatsapp groups dedicated to residents working in hospitals and primary care facilities of the ministry of health all over Egypt as well as social media groups whose members were residents in university hospitals including Cairo, Ain Shams and Alexandria Universities (located in northern Egypt), Suez Canal and Portsaid Universities (located in East Egypt), Sohag and Assiut Universities (located in the South of Egypt). In addition, participants who completed the survey were asked to share the link with their colleagues. We included medical residents of both genders, who have been on the job at least 3 months, so that they would have enough experience to answer the questionnaire. The study excluded those who were known to have a psychiatric diagnosis, or chronic pain disorders (fibromyalgia, Rheumatoid Arthritis, chronic back pain, etc.) before entering the residency program, to ensure that the musculoskeletal pain reported by the participants occurred after starting their residency.

#### Sample size determination

We used G*Power application version 3.1.9.7 using Correlation bivariate normal model method, with significance level 95%, a power of 80%, using an effect size (correlation coefficient) 0.3 (medium correlation). Calculation revealed sample size of 84, adding 20% (17 subjects) counting for non-response rate, the sample size was 101 [33].

#### Ethical considerations

Ethical Approval: The study was approved by the research ethics committee of the Faculty of Medicine, Suez Canal University, Egypt (Reference: 4485#),

Informed Consent: The Google forms link included an information page about the purpose and methods of the study. An informed consent was secured by clicking the “agree to participate” button at the end of the information sheet.

#### Study tools

We used an online questionnaire that consisted of 4 sections:

##### Section 1: socio-demographic data

These included age, sex, marital status, type of workplace, medical specialty, level of postgraduate education, working hours, and employment status.

##### Section 2: musculoskeletal pain

A. Widespread Pain Index

For assessment of musculoskeletal pain, we used the Widespread Pain Index (WPI) which is employed in the diagnosis of chronic pain syndromes. The index was originally developed as part of the 2010 American College of Rheumatology classification criteria for Fibromyalgia [34]. Since then, it has been used more widely to assess widespread pain in studies of general pain conditions [35]. The index was shown to be highly sensitive and accurate in assessment of widespread chronic pain [36, 37]. The WPI includes a list of 19 possible painful body areas. It gives a total score that ranges from 0 to 19 [35]. In addition to identifying separate pain sites, the index can also identify subjects with widespread pain. Those are subjects with a minimum of 4 painful sites [38].

B. Patient reported outcomes measurement information – pain interference (PROMIS-PI) short form [39]

The PROMIS-PI short form was developed using Item Response Theory [39]. The 6 items of the short form were chosen from the original PROMIS- PI 41 item bank. Each item is scored on a 5-point Likert scale ranging from “not at all” to “very much”, except for the last item which enquires about socializing with others and ranges from “never” to “always”. The questionnaire has good construct validity and precision [40].

##### Section 3: violence and harassment

This section was adopted from previous research [41, 42]. Al-Shafaee et al. (2013) used this set of items to quantify the frequency of different forms of violence, including verbal abuse, physical abuse, sexual harassment, and academic misuse of power in a cohort of medical interns. The same items were used for the purpose of quantifying violence in this study. A 7- point Likert Scale was used with score assigned to each response where “Not at all” =0; “Less than once a month” =1; “Once a month” =2; Few times a month” =3; “Once a week” =4; and “Few times a week” =5; and “Everyday” =6.

We calculated a unique index for each type of abusive behaviors by adding the total score of its component divided by the total number of items multiplied by 6. A total abuse index was calculated by adding the indices for the 4 types of abusive behaviors (verbal abuse, physical abuse, sexual abuse or harassment and academic misuse of power).

##### Section 4: workplace climate

This section assessed the residents’ perception towards workplace environment regarding the psychosocial aspect and the extent of feeling secure in their workplace. For this section, we used 2 questionnaires.

A. Psychosocial Safety Climate (PSC) Questionnaire [43]:

This questionnaire is a 12-item short instrument that is used to measure the main four domains of safety climate, namely Senior management commitment, Management priority, Organizational participation, and Organizational communication with employees regarding their psychosocial safety and well-being. The items are measured using a 5-point Likert format ranging from 1 (strongly disagree) to 5 (strongly agree). Total scores range from 12 to 90, and are classified as low-risk (≥ 41), medium risk [37–40], high risk [26–36] and very high risk (< 26) [43]. This tool can be used for different occupations and within organizations, with accepted internal consistency (Cronbach’s α of 0.94 for the 12 items) as shown in previous research [44].

B. Sexual harassment climate questionnaire [45, 46]

This section assessed the residents’ perception about the psychological climate for sexual harassment through a questionnaire composed of 9 items inquiring about two main topics. The first is their risk perception to report an incident of sexual harassment (3 questions), while the second topic inquiries about whether they think the report will be taken seriously within the organization (6 questions), with Cronbach’s α for these items ranges from 0.59 to 0.7 for internal consistency as shown in prior research [45, 46]. The items were measured using a 5-point Likert format ranged from 1 (strongly disagree) to 5 (strongly agree), with higher scores indicating a greater intolerance of sexual harassment.

### Statistical analysis

Data was analyzed using the Statistical Package for Social Sciences (SPSS) version 23. Quantitative variables were presented as either mean ± standard deviation or median and interquartile range; qualitative data were presented as frequency and percentage. We tested association of demographic and work-related variables with the widespread pain index and abuse indices using either Mann-Whitney or Kruskal- Wallis tests with pairwise comparisons for significant differences performed by Dunn Bonferroni test. Spearman correlation was used to test the relation between WPI, duration of residency, abuse index and psychosocial safety climate score. Finally, we performed a backward linear regression analysis for predictors of WPI including abuse index. Factors entered into the model included those showing significant statistical relation to WPI in univariate statistical analysis. *P*-value < 0.05 was considered statistically significant.

## Results

The study included 101 medical residents working in Egyptian hospitals. About (54.5%) of the participants were older than 28 years and females were more represented than males (86.1%). Regarding occupational characteristics, 93.1% of the participants worked in the public sector while only 6.9% worked in the private sector. About three quarters (75.2%) of the study participants worked both morning and night shifts. Work shift duration was mostly 12 hours (37.6%) followed by 6 hours (34.7%). Family medicine residents were the most represented specialty (23.8%) while obstetrics and gynecology were the least frequent specialty (2%). Other specialties included clinical pathology, emergence medicine, anesthesia, and intensive care unit residents (13.9% each). Most participants held a master’s degree (47.5%), and about 13.9% finished their medical doctorate. More than one third (38.6%) of the participants were fresh graduates. The mean duration of residency was 3.1 ± 2.1 ranging from 0.5 to 12 years. Table 1 shows the sociodemographic characteristics of the study participants.

**Table 1:** Basic characteristics of study participants

Demographic characteristics	No. (%)
Age	
25-	13(12.9%)
27–28	33(32.7%)
> 28	55(54.5%)
Gender	
Females	87(86.1%)
Males	14(13.9%)
Marital status	
Single	42(41.6%)
Married	58(57.4%)
Divorced	1(1.0%)
**Occupational characteristics**	
Working place	
Private	7(6.9%)
Public	94(93.1%)
Working hours	
Morning shift	25(24.8%)
Both Morning and Evening shifts	76(75.2%)
Shift duration	
6 hours	35(34.7%)
12 hours	38(37.6%)
18 hours	7(6.9%)
24 hours	21(20.8%)
Specialty	
Clinical pathology	14(13.9%)
EM, Anesthesia and ICU	14(13.9%)
Family medicine	24(23.8%)
Medicine	13(12.9%)
Neuropsychiatry	7(6.9%)
Obstetrics /gynecology	2(2.0%)
Pediatrics	12(11.9%)
Radiology	6(5.9%)
Surgery	9(8.9%)
Highest level of education	
Fresh graduate	39(38.6%)
Master	48(47.5%)
Doctorate	14(13.9%)
Duration of residency	
Mean ± SD	3.1 ± 2.1
Median, range	3, 0.5–12

*EM* Emergency Medicine, *ICU* Intensive Care Unit

### Measurement of physical pain and pain interference

**Fig. 1:**
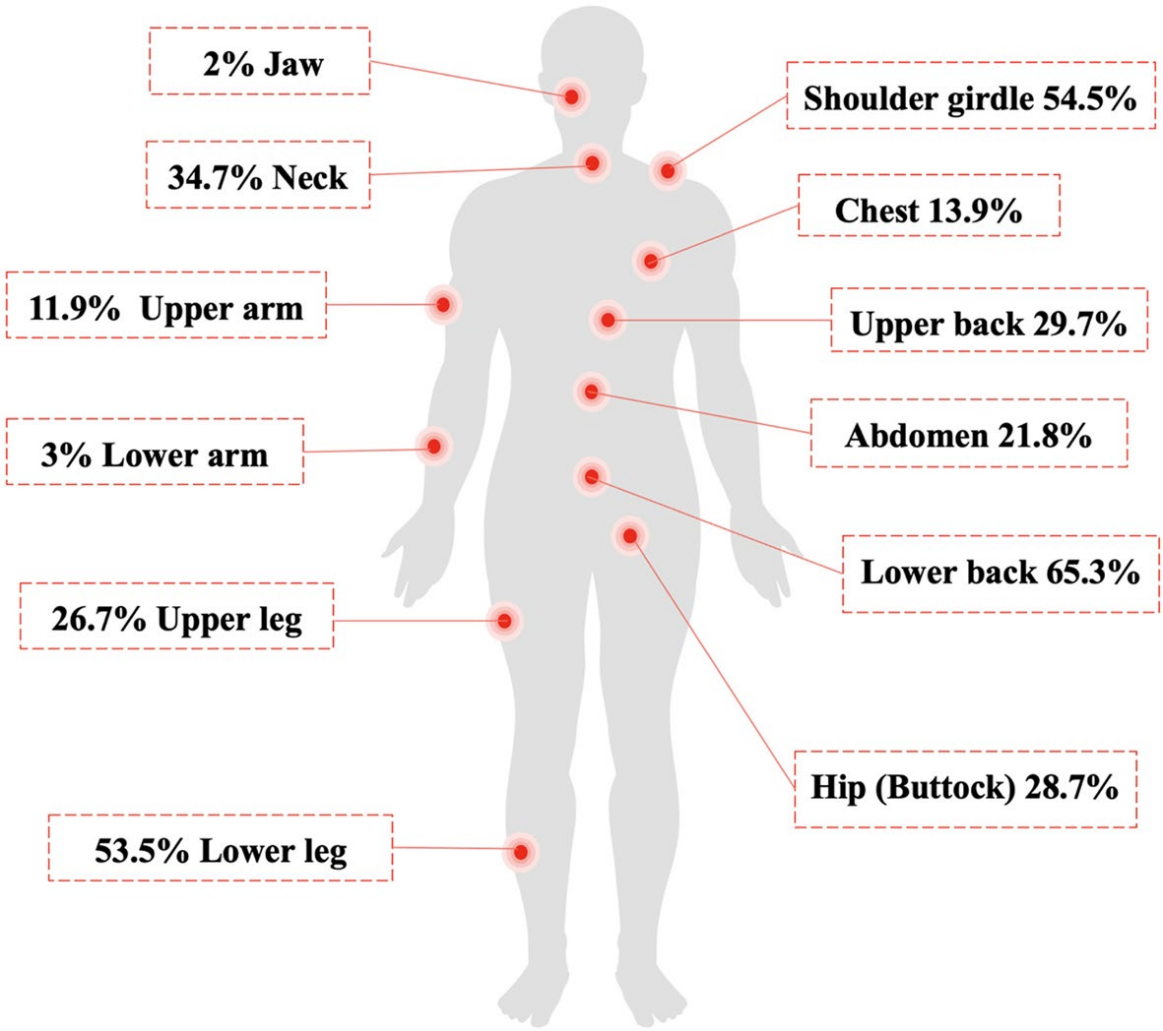
Frequency of pain sites reported by study participants

All participants had at least one painful site (range 1 to 11, the maximum possible number of pain sites is 19). The mean widespread pain index was 3.5 ± 2.4, with a median of 3.0 and IQR of 3.5 (These results are not tabulated). Forty of the 101 participants (39.6%) satisfied the definition of having widespread pain by having 4 or more pain sites. The most reported site of pain was the lower back (65.3%), and the least reported site was the jaw (2%). Frequency for each pain site is shown in Fig. 1. Regarding pain interference, the highest percentage for interference was recorded for the item “Pain a little bit interfered with day-to-day activities (34.7%), while the lowest percentage was recorded for “Pain interference very much with day-to-day activities” and “Pain interfered very much with enjoyment and recreational activities” (8.9% each). PROMIS – PI scores for pain interference with activities as reported by the study participants are presented in Table 2.

**Table 2:** Pain interference with activities (PROMIS – PI) during the preceding 7 days (*n* = 101)

	Not at all	A little bit	Somewhat	Quite a bit	Very much
1. Pain interfered with life enjoyment	22(21.8%)	31(30.7%)	23(22.8%)	14(13.9%)	11(10.9%)
2. Pain interfered with ability to concentrate	23(22.8%)	31(30.7%)	24(23.8%)	13(12.9%)	10(9.9%)
3. Pain interfered with day-to-day activities	21(20.8%)	35(34.7%)	24(23.8%)	13(12.9%)	8(8.9%)
4. Pain interfered with enjoyment of recreational activities	20(19.8%)	29(28.7%)	28(27.7%)	15(14.9%)	9(8.9%)
5. Pain interfered with doing tasks away from home (e.g., getting groceries, running errands)	19(18.8%)	25(24.8%)	24(23.8%)	18(17.8%)	15(14.9%)
	**Never**	**Rarely**	**Sometimes**	**Often**	**Always**
6. Pain kept from socializing with others	11(10.9%)	24(23.8%)	43(42.6%)	19(18.8%)	4(4.0%)

### Frequency and forms of abuse

The most common reported verbal abuse was “shouting and yelling” being reported daily by 26.7% and few times a week by 24.8% of participants. The most common form of harassment was discrimination on the basis of age, gender or religion occurring daily in 6.9% of participants and a few times per week to 5% of participants. Regarding academic abuse, shifting of responsibilities (i.e., where more responsibilities are unjustly assigned to the resident) was the most common form reported by 12.9% on daily basis and 11.9% a few times per week (results are not tabulated).

### Workplace psychological climate and well-being

According to the PSC score, 36.6% of the residents perceived their work environment to be high-risk of PSC and 35.6% scored very high-risk PSC. The category with the highest mean was Senior Management Commitment, and the category with the lowest score was Organizational Communication with Employees. Workplace psychological climate total and category scores are presented in Table 3.

**Table 3:** Workplace psychosocial safety climate scores among study participants (*n* = 101)

Item	Mean ± SD	Median	IQR
**Psychosocial Safety Climate total score**	29.0 ± 11.3	27	19–37
Senior Management Commitment	7.8 ± 3.2	8	5–10
Management Priority	7.4 ± 3.3	7	4.5–10
Organizational Participation	7.0 ± 3.0	7	3.5–9
Organizational Communication with Employees regarding their Psychosocial safety and Well-being	6.7 ± 3.1	7	5–9

*IQR* interquartile range

### Sexual harassment climate and reporting of sexual abuse

Almost half of the study participants (47.6%) agreed (either agreed or strongly agreed) that a sexual harassment complaints would be thoroughly investigated. Less than half (40.6%) thought they would be comfortable reporting a sexual harassment complaint. However, a similar percentage (41.6) perceived that individuals who sexually harass others get away with it. Additionally, about one third (31.6%) thought it would be risky to file a sexual harassment complaint (results are not tabulated).

**Fig. 2:**
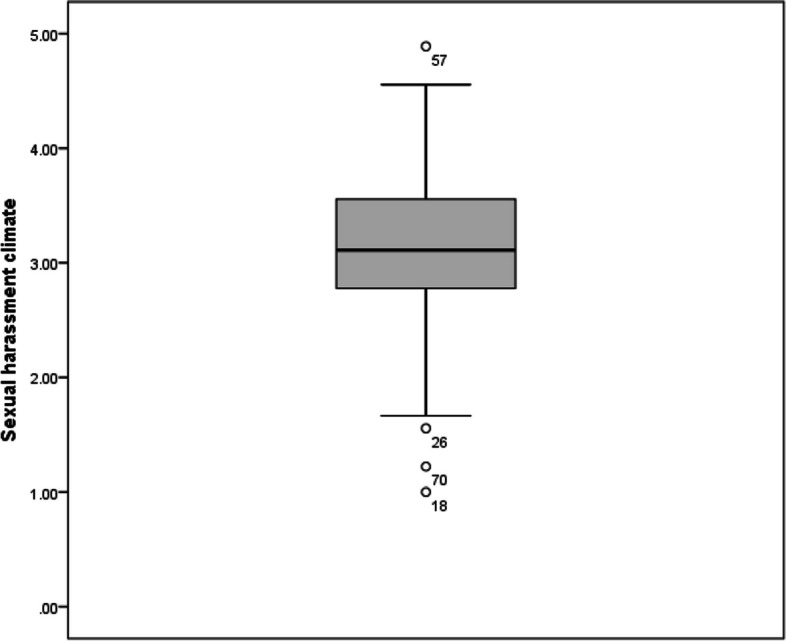
Sexual harassment climate score among studied residents (*n* = 101)

**Fig. 3:**
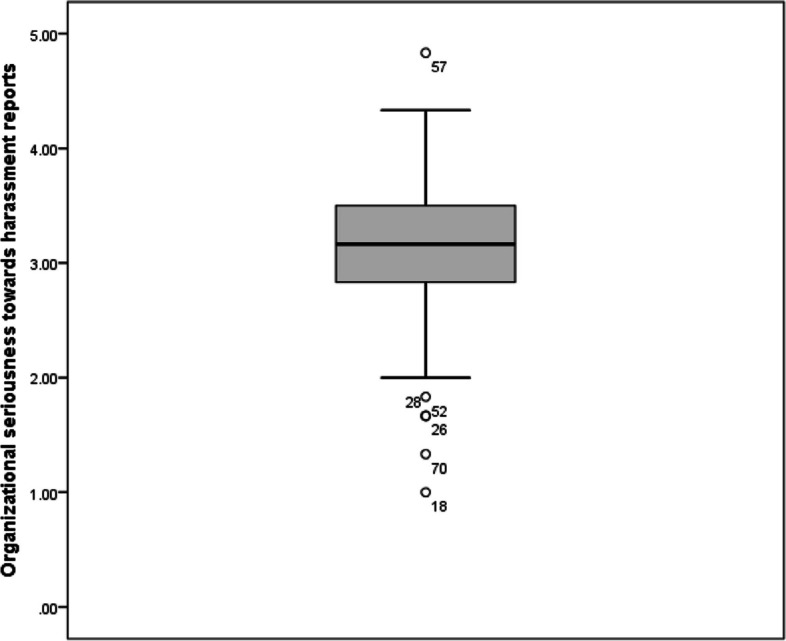
Organizational seriousness towards harassment reports as reported by studied residents (*n* = 101)

**Fig. 4:**
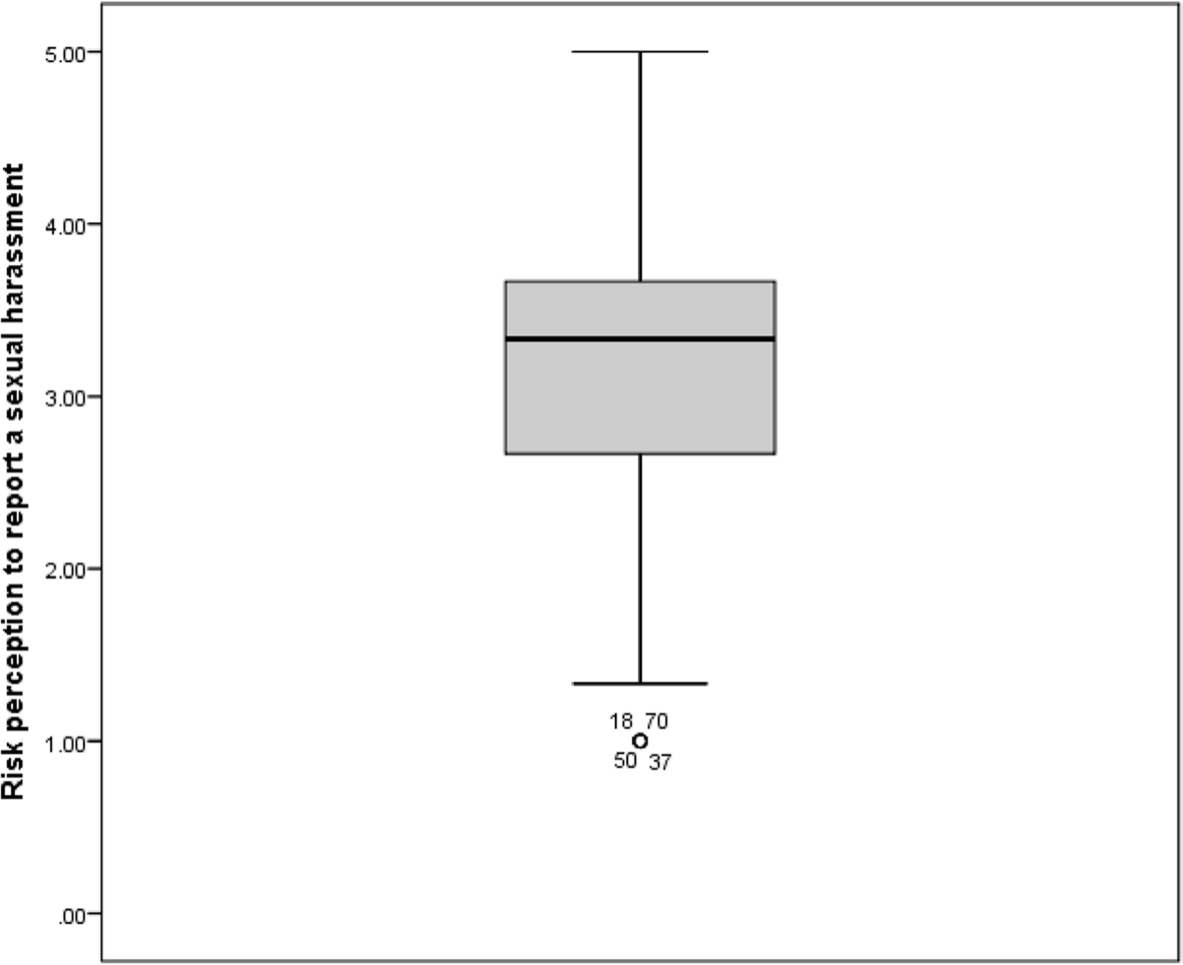
Risk perception to report a sexual harassment among studied residents (*n* = 101)

*Risk perception to report a sexual harassment* was assessed by scoring three questions about how safe the resident feels reporting a sexual harassment. The mean score for risk perception to report a sexual harassment was 3.2 ± 1.0 with a median of 3.3 and interquartile range (IQR) of 1.2. On the other hand, *seriousness of organization towards report* was assessed by four questions about how the organization will deal with a reported sexual harassment. The mean score for organizational seriousness towards harassment reports was 3.1 ± 0.7 with a median of 3.2 and IQR of 0.7. The overall mean sexual harassment climate score was 3.1 ± 0.7 with a median of 3.1 and IQR of 0.8 (Figs. 2, 3 and 4).

### Associations of WPI with demographic and work-related characteristics

Widespread pain index did not show any significant relation to demographic and occupational characteristics of the study participants (Table 4).

**Table 4:** Association between WPI and demographic and work-related characteristics of the study participants (*n* = 101)

Basic characteristics	Widespread pain index	
	Median (IQR)	*P* value
**Demographic characteristics**		
Age		
25-	3.0(3.5)	0.629^§^
27–28	3.0(4.5)	
> 28	3.0(3.0)	
Gender		
Females	3.0(3.0)	0.377^¥^
Males	2.5(3.3)	
Marital status		
Single	3.0(3.0)	0.499^§^
Married	3.0(4.0)	
Divorced	4.0	
**Occupational characteristics**		
Working place		
Private	3.0(2.0)	0.924^¥^
Public	3.0(4.0)	
Working shifts		
Morning shift	2.0(3.0)	0.095^¥^
Both Morning and Evening shifts	3.0(3.0)	
Shift duration		
6 hours	2.0(3.0)	0.191^§^
12 hours	3.0(3.3)	
18–24 hours	3.0(4.0)	
Specialty		
Clinical pathology	2.0(2.3)	0.084^§^
ER, Anesthesia and ICU	4.0(3.5)	
Family medicine	3.0(3.3)	
Medicine	2.0(3.0)	
Neuropsychiatry	3.0(2.0)	
Obstetrics /gynecology	4.0(2.0)	
Pediatrics	2.5(2.8)	
Radiology	4.5(6.8)	
Surgery	4.0(3.0)	
Education		
Fresh graduate	3.0(4.0)	0.219^§^
Master’s	2.0(3.0)	
Doctorate	4.0(2.5)	

^¥^ Mann-Whitney test was used, ^§^ Kruskal-Wallis test was used

### Associations of workplace PSC, sexual harassment index and abuse index with demographic and work-related characteristics

Workplace safety climate scores showed significant difference between participants in relation to their marital status where married participants had a median of 29.5 and IQR of 15.5, single residents (median = 27, IQR = 13.3) and the divorced residents had a median score of 12 (*p* = 0.047). Pairwise comparison between marital statuses did not show any significant difference between pairs. There was no significant difference in the PSC, sexual harassment and abuse index scores in relation to the remaining demographic characteristics. On the other hand, workplace PSC was significantly higher among those working morning shift only (median = 35, IQR = 20.5) than those working both morning and night shifts (median = 27, IQR = 14.8) (*p* = 0.016). There was a significant relation between residents’ specialty and workplace PSC; the highest score was recorded among emergency medicine, intensive care unit (ICU) and Anesthesia residents (median = 31, IQR = 16.3) followed by family medicine (median = 30, IQR = 19.3) and clinical pathology (median = 30, IQR = 12.5); the least score was recorded for obstetrics and gynecology residents (median = 12.5, IQR = 0.5) (*p* = 0.047). Pairwise comparisons did not show statistically significant differences between different specialties as regard workplace PSC score. Regarding the abuse index, there was a significant difference between residents working morning shifts only (median = 0.7, IQR = 0.7) and those working both morning and night shifts (median = 1, IQR = 1) (*p* = 0.018). Additionally, there was a significant difference in Abuse score between residents working 6-hour shifts (median = 31.0, IQR = 17.0), 12-hour shifts (median = 27.5, IQR = 16.5), and 18–24-hour shifts (median = 25.5, IQR = 12.8) (*p* = 0.034). Pairwise comparison showed significant difference in Abuse index between residents working 6-hours shifts and those working 12-hour shifts (*p* = 0.021), and 18–24-hours shifts (*p* = 0.031). Once more, there were significant differences across specialties (*p* < 0.01), where the highest scores belonged to obstetrics and gynecology residents (Median = 1.9, IQR = 0.7), followed by Surgery (Median = 1.3, IQR = 1) (Table 5). Pairwise comparisons showed significant difference in Abuse index between family medicine residents and each of surgery residents (*p* = 0.023) and residents of EM, Anesthesia and ICU specialty (*p* = 0.001).

**Table 5:** Associations between demographic and work-related characteristics of the study participants and Workplace psychological safety climate; Sexual harassment climate scores; and Abuse index

	Workplace PSC scores		Sexual Harassment Climate score		Abuse Index	
	Median (IQR)	*P* value	Median (IQR)	*P* value	Median (IQR)	*P* value
**Demographic characteristics**						
Age						
25-	27(16)	0.118^§^	3.0(1.1)	0.154^§^	0.6(0.1–1.6)	0.682^§^
27–28	27(15)		3.2(1.0)		0.7(0.5–1.4)	
> 28	30(17)		3.1(0.8)		0.9(0.5–1.3)	
Gender						
Females	27.0(18.0)	0.612^¥^	3.1(0.8)	0.581	0.8(0.4–1.3)	0.198^¥^
Males	28.5(15.5)		3.1(0.5)		1.1(0.7–1.4)	
Marital status						
Single	27.0(13.3)	0.047*^§^	3.1(0.8)	0.112^§^	0.9(0.5–1.5)	0.070^§^
Married	29.5(15.5)		3.2(0.8)		0.7(0.3–1.3)	
Divorced	12.0		2.7		1.8	
**Work-Related Characteristics**						
Working place						
Private	31.0(14.0)	0.462^¥^	3.0(1.3)	0.851^¥^	0.7(1.4)	0.894^¥^
Public	27.0(18.0)		3.1(0.8)		0.9(0.9)	
Working shifts						
Morning shift	35.0(20.5)	0.016*^¥^	3.1(0.6)	0.862^¥^	0.7(0.7)	0.018*^¥^
Both Morning and Evening shifts	27.0(14.8)		3.1(0.8)		1.0(1.0)	
Shift duration						
6 hours	31.0(17.0)	0.068^§^	3.2(0.5)	0.262^§^	0.7(0.9)	0.034*^§^
12 hours	27.5(16.5)		3.2(1.1)		1.0(0.9)	
18–24 hours	25.5(12.8)		3.0(0.8)		0.9(1.0)	
Specialty						
Clinical pathology	30.5(18.0)	0.047*^§^	3.6(0.9)	0.172^§^	0.7(0.8)	0.001*^§^
EM, Anesthesia and ICU	31.0(16.3)		3.1(1.0)		1.1(1.1)	
Family medicine	30.5(19.3)		3.3(0.5)		0.4(0.8)	
Medicine	26.0(21.0)		3.1(0.5)		0.8(0.8)	
Neuropsychiatry	22.0(12.0)		2.7(0.8)		1.0(0.7)	
Obstetrics /gynecology	12.5(0.5)		3.8(0.6)		1.9(0.7)	
Pediatrics	26.5(13.5)		3.1(1.3)		0.7(1.0)	
Radiology	29.0(14.8)		2.8(1.0)		0.9(1.1)	
Surgery	27.0(10.5)		3.0(0.9)		1.3(1.0)	
Education						
Fresh graduate	27.0(14.0)	0.184^§^	3.1(0.8)	0.180^§^	0.8(0.4–1.4)	0.900^§^
Master’s	28.5(22.0)		3.2(0.8)		1.0(0.5–1.3)	
Doctorate	36.0(17.0)		2.9(0.8)		0.8(0.5–1.2)	

*PSC* Psychological Safety Climate, * Statistically significant at *p* value < 0.05, ^¥^ Mann-Whitney test was used, ^§^ Kruskal-Wallis test was used

### Correlation between WPI and workplace PSC and abuse index

Widespread pain index showed a statistically significant weak negative correlation with workplace PSC score (rho = − 0.272, *p* = 0.006). Conversely, there was a statistically significant weak positive correlation between WPI and the calculated total abuse index (rho = 0.305, *p* = 0.002). In addition, a weak non-significant correlation was found between WPI and sexual harassment climate score (Table 6).

**Table 6:** Correlation between Widespread pain index and duration of work; Workplace psychosocial safety climate score; and Sexual harassment climate score

	Widespread Pain Index	
	Spearman’s rho Correlation Coefficient	*P* value
**Duration of residency**	0.041	0.683
**Workplace Psychosocial Safety Climate Score**	−0.272	0.006*
**Sexual Harassment Climate Score**	−0.182	0.068
**Abuse Index**	0.305	0.002*

* Statistically significant at *p* value < 0.05

### Predictors of WPI score

Regression analysis of predictors of WPI was performed. Results are presented in Table 7, where factors entered into the model were duration of residency, sexual harassment climate score, workplace psychosocial safety climate and abuse index. Abuse index was found to be the only predictor of WPI (*p* = 0.002).

**Table 7:** Regression analysis of predictors of Widespread pain index

	Unstandardized Coefficients		Standardized Coefficients	t	*P* value	95% Confidence Interval for B	
	B	Std. Error	Beta			Lower Bound	Upper Bound
(Constant)	2.433	0.393		6.197	< 0.001*	1.654	3.212
**Abuse Index**	1.100	0.342	0.307	3.214	0.002*	0.421	1.780

* Statistically significant at *p* value < 0.05

## Discussion

We aimed to explore the relationship between workplace factors, namely workplace violence (WPV) and musculoskeletal pain among Egyptian medical residents. Results of the current study confirm the presence of an association between the two phenomena. In addition, our results show a high prevalence of musculoskeletal pain among residents, where about 40% of our sample experienced widespread musculoskeletal pain, with low back pain being the most prevalent pain site. Previous studies worldwide report high prevalence rates of musculoskeletal symptoms among physicians [5, 47], with reported prevalence of low back pain as high as 68% [2]. Previous studies in Egypt, studies show similar prevalence rates of musculoskeletal pain among physicians [9, 48]. With the alarming increase of prevalence of chronic pain among the general population and among physicians, factors that contribute to such pain warrant in-depth investigation and prompt preventive actions [49].

Models that explain work-related musculoskeletal pain have evolved over time from ones that recognize mechanical factors as the sole determinant for musculoskeletal complaints to those that appreciate the complex nature of pain as a biopsychosocial phenomenon [50–52]. Despite these recent models, most studies still focus on either physical (including structural, ergonomic and biomechanical factors) and psychological factors, with less focus on the social and work-related components [53, 54]. Previous studies have explored the effects of job strain [55], long working hours [56], and overexertion at work [57], while studies that focus on WPV as a risk factor for chronic pain among physicians are few.

Our results show that medical residents in Egypt work in a challenging environment, where the risks of violence, sexual harassment and abuse are high, and the sense of safety and perception of support against such violence are low. Previous studies conducted in Egypt also show high a high prevalence of violence. Mahmoud et al. (2022) reported that in a sample of 445 physicians, 82.5% reported exposure to violence [58]. Other studies reported prevalence rates ranging from 59.7 and 72.6% [59–61]. In the current study, residents working night shifts, and those working 12–24 hour-shifts were found to be more exposed to violence compared to physicians who work morning shifts only and 6–12-hour shifts. Similar results were found by Mahmoud et al. (2022), where exposure to violence was associated with higher working hours/week, as well as number of staff/work setting. A higher work load can lead to physician exhaustion, coupled with long patient waiting time, which could explain the increase in incidences of violence [58]. As with our results, previous studies also show that Emergency physicians are more prone to violence than other specialties [59, 62].

Regarding sexual harassment and reporting of abuse, we found that more than half of our sample would not feel comfortable reporting harassment complaints. There is a similar global lack of reporting of incidences of violence and sexual harassment, and a subsequent state of inaction which will only serve to aggravate the consequences of WPV [13, 63]. Lack of reporting is attributed to absence of trust in the reporting system, lack of action from the authorities [20, 64]. One study indicated that the reason for not reporting violence was that healthcare workers have become accustomed to violence, to the extent that they no longer report it [31].

Our results show a significant, although weak, correlation between the degree and magnitude of workplace abuse and musculoskeletal pain. Similar results were reported among nurses and other clinical staff [10, 65, 66]. Moreover, a dose-response relationship was found between incidences of assault and musculoskeletal pain [11]. Additionally, women exposed to workplace violence were found to show significantly higher pain levels than other workers [67]. The relationship between violence and physical pain is somewhat complex. Studies show that the perception of pain, the transition to chronicity and the degree of pain interference with the quality of life are not driven merely by the initial injuries causing the pain (i.e. trauma or sprain), but by a set of underlying psychological processes and environmental triggers that account for persistence, chronicity and disability [68]. The continuous or recurrent exposure to violence, including physical and emotional traumas, serves as triggers that exacerbate underlying vulnerabilities such as distress and anxiety, and exhaust protective mechanisms, such as coping and resilience [69].

Regarding workplace safety climate, our results show a negative correlation between perception of psychological safety and presence of musculoskeletal pain. A somewhat similar finding was reported by Zadow et al. who found that low workplace psychological safety climate was strongly associated with low psychological health and high incidence of work injuries in healthcare workers [70]. Specifically, workplace safety climate is the result of the different dynamics of the various levels of an organization. Such dynamics include how decisions are made, tasks are assigned, goals are set, and mistakes are dealt with. These organizational processes could ultimately decide the workloads, job demands, degree of work engagement and, in part, the psychological health of workers [71], and ultimately the safety and satisfaction of the end users (i.e. patients and their relatives) [72]. Unfortunately, studies report that the workplace safety climate in healthcare report various degrees of poor safety climates, with subsequent negative effects on job stress, low job satisfaction and high job-quitting intent [73–75].

Regarding sexual harassment, we found no correlation between musculoskeletal pain and sexual harassment climate score. Although to our knowledge, no studies have reported an association between sexual harassment and pain among physicians, studies on the general population show a relationship between exposure to sexual abuse and chronic pain [76, 77]. The lack of association in our study could be related to the tool we used, which enquires about reporting of sexual harassment, rather than the occurrence of actual incidences of sexual abuse at the workplace.

Finally, our study found that exposure to abuse predicts physical pain among our sample of physicians. This alarming finding was also reported in nurses, where exposure to violence was found to predict physical symptoms, including back pain, upper body pain and lower extremity pain. Moreover, a change in the exposure to violence, whether by an increase or a decrease, led to a change in physical symptoms in the same direction [67]. Similarly, Miranda et al. found that a combination of poor workplace safety and violent assaults increased the risk of widespread pain among nursing home workers [11]. Unlike exposure to abuse, workplace safety climate did not predict physical pain in our study, but did show a significant negative correlation with widespread pain.

The current study supports the presence of an association between WPV and musculoskeletal pain among physicians. However, several limitations should be kept in mind when interpreting results of the current study. First, the inquiry about incidences of abuse during the whole period of residency may raise the possibility of recall bias, which could lead to either over or under-reporting of forms of abuse. Second, the convenient sample and the absence of a sampling frame did not allow for calculation of a response rate. Also, volunteer bias could have occurred, where physicians who were subjected to violence might have been more eager to participate in the study. Third, the use of the widespread pain index allowed for quantification of the number of sites of pain, however, it did not give an indication for the severity of pain in each site. Finally, due to the cross-sectional design of the study, we were unable to examine whether a temporal relationship exists between variables.

## Conclusions

The current study provides evidence of an association between WPV and widespread musculoskeletal pain among physicians. The study also shows that both WPV and musculoskeletal pain are alarmingly prevalent among medical residents in Egypt. These two phenomena should be the targets of preventive strategies that enhance workplace safety climate and physical wellbeing of physicians to reduce incidences of violence and all its hazardous effects, including musculoskeletal pain.

## Data Availability

The dataset generated and/or analyzed during the current study are available from the corresponding author upon reasonable request.

## Abbreviations

ICU: Intensive care unit
PROMIS-PI: Patient reported outcomes measurement information – pain interference
PSC: Psychosocial safety climate
WPI: Widespread pain index
WPV: Workplace violence
